# Hormonal Intensification Should Start at the Low-risk Stage in Metastatic Prostate Cancer

**DOI:** 10.1016/j.euros.2022.05.015

**Published:** 2022-09-28

**Authors:** Seyed Behzad Jazayeri, Lauren Folgosa Cooley, Abhishek Srivastava, Neal Shore

**Affiliations:** a Department of Urology, University of Florida, Jacksonville, FL, USA; b Department of Urology, Northwestern University, Chicago, IL, USA; c Genesis Care, Myrtle Beach, SC, USA

**Keywords:** Prostate cancer, Abiraterone, Docetaxel, Enzalutamide, Apalutamide, Darolutamide

## Abstract

The treatment landscape for metastatic hormone-sensitive prostate cancer (mHSPC) has dramatically evolved. Monotherapy androgen deprivation therapy (ADT) with testosterone suppression alone is no longer the standard of care as multiple global phase 3 trials of different combinatorial strategies have been clinically and statistically successful and the combinations have been incorporated into guidelines on advanced prostate cancer. For appropriate patients, clinicians should consider combining ADT with docetaxel or an androgen receptor pathway inhibitor, or possibly with both. Shared patient-physician decision-making mandates a review of the level 1 evidence supporting the optimization and intensification of combination therapy for patients with mHSPC. Here we discuss the evidence underscoring intensification strategies as the standard of care for low-volume, low-risk mHSPC.

**Patient summary:**

We discuss treatment strategies for men with metastatic prostate cancer. Combinations of androgen deprivation therapy (ADT) and drugs that inhibit the androgen receptor pathway are superior to ADT alone and prolong survival in patients with metastatic hormone-sensitive prostate cancer.

The terminology for low-volume and low-risk staging of prostate cancer (PC) stems from two landmark trials, LATITUDE [1] and CHAARTED [2], which analyzed the efficacy of abiraterone acetate plus prednisone (AAP) in prolonging overall survival (OS) in men with metastatic hormone-sensitive PC (mHSPC). High risk was defined in the LATITUDE trial as meeting two of the following three criteria: a Gleason score of ≥8, three or more bone lesions, and the presence of visceral metastasis [1]. In the CHAARTED trial, high-volume disease was defined as the presence of visceral metastasis and/or four or more bone lesions, with one or more outside the vertebral bodies or pelvic bones [2]. Following these two successful trials in mHSPC, subgroup analyses comparing low-risk versus high-risk risk and low-risk versus high-volume PC have been performed for the well-designed and -conducted, multiarm, multistage STAMPEDE trial platform, along with other global trials evaluating combination therapy involving androgen deprivation therapy (ADT) with either enzalutamide (ENZA) or apalutamide (APA). AAP, ENZA, and APA have received international regulatory approval for combination with ADT for low-risk, low-volume mHSPC, as well as for patients with high-risk and/or high-volume mHSPC [2]. A summary of the published trials and oncologic outcomes for men with low-risk, low-volume mHSPC treated with hormonal intensification strategies is presented in Table 1 and Figure 1.

**Table 1:** Summary of trials and oncologic outcomes for men with metastatic hormone-sensitive prostate cancer

	Phase	Intervention	Control arm	Total patients	LV mHSPC, *n* (%)	LV definition^a^	Primary endpoint(s)
**Abiraterone**							
STAMPEDE NCT00268476 post hoc analysis	3	Abiraterone (1000 mg) plus prednisone (5 mg) + ADT (trial arm G)	ADT alone (trial arm A)	2751 (901 included in analysis)	402 (14.6%)	CHAARTED criteria	OS: HR 0.64 (95% CI 0.42–0.97) 3-yr OS: 83% vs 77% FFS: HR 0.26 (95% CI 0.19–0.36) 3-yr FFS: 74% vs 32%
**Enzalutamide**							
ARCHES NCT02677896	3	Enzalutamide (160 mg daily) + ADT	Placebo + ADT	1150	423 (36.8%)	CHAARTED criteria	rPFS: HR 0.25 (95% CI 0.14–0.46)
ENZAMET NCT02446405	3	Enzalutamide (160 mg daily) + ADT	NSAA + ADT	1125	272 (24.2%)	CHAARTED criteria	OS: HR 0.43 (95% CI 0.26–0.72)
ENZAMET NCT02446405 post hoc analysis	3	Enzalutamide (160 mg daily) + ADT	NSAA + ADT	1125	205 (18.2%)	– M0 at diagnosis – CHAARTED criteria	OS: HR 0.40 (95% CI 0.16–0.97) 3-yr OS: 92% vs 83%
**Apalutamide**							
TITAN NCT02489318	3	Apalutamide (280 mg daily) + ADT	Placebo + ADT	1052	392 (37.2%)	CHAARTED criteria	OS: HR 0.52 (95% CI 0.35–0.79)

LV = low volume; mHSPC = metastatic hormone-sensitive prostate cancer; ADT = androgen deprivation therapy; NSAA = nonsteroidal antiandrogen; OS = overall survival; rPFS = radiographic progression-free survival; FFS = failure-free survival; HR = hazard ratio; CI = confidence interval.

a CHAARTED criteria for LV versus high-volume disease: high-volume disease is defined as the presence of visceral metastasis and/or four or more bone lesions with one or more outside of the vertebral bodies or pelvic bone.

**Fig. 1:**
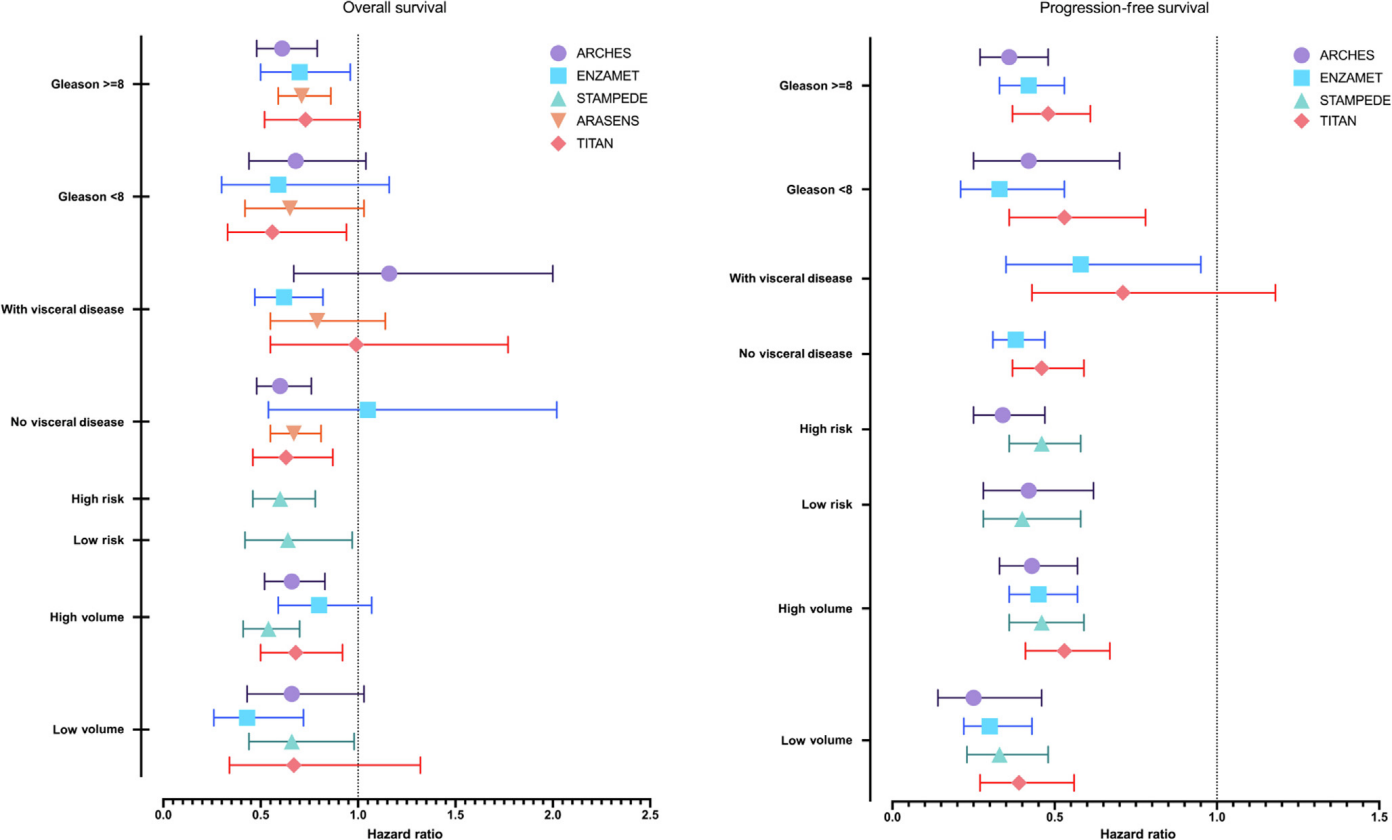
Forest plot of hazard ratios for overall survival and progression-free survival in subgroups from the ARASENS, ARCHES, ENZAMET, STAMPEDE, and TITAN trials.

The STAMPEDE trial assessed the benefit of early initiation of AAP in addition to ADT in men with locally advanced or metastatic PC starting ADT for the first time [3]. A post hoc analysis [4] of STAMPEDE evaluated men with low-volume, low-risk mHSPC defined according to CHAARTED [2] and LATITUDE [1] criteria. The group with low-risk mHSPC who received AAP had better OS (hazard ratio [HR] 0.66, 95% confidence interval [CI] 0.44–0.98) and failure-free survival (FFS; HR 0.25, 95% CI 0.17–0.33) in comparison to ADT alone [4]. In addition, men with low-risk mHSPC who received AAP had higher skeletal-related event (SRE)-free survival (HR 0.31, 95% CI 0.18–0.54), longer progression-free survival (PFS; HR 0.33, 95% CI 0. 23–0.48), and lower PC-specific mortality (HR 0.51, 95% CI 0.31–0.84) [4].

The ARCHES trial [5] compared ENZA plus ADT to ADT alone for men with mHSPC. ENZA improved radiographic PFS in the subgroups of men with low-volume (HR 0.25, 95% CI 0.14–0.46) and low-risk disease (HR 0.42, 95% CI 0.28–0.62). Men receiving ENZA also had prolonged OS (HR 0.66, 95% CI 0.52–0.81), although subgroup-specific data have not been reported [5], [6]. In the ENZAMET trial, men with mHSPC were randomized to receive ENZA plus ADT or a nonsteroidal antiandrogen drug (bicalutamide, nilutamide, or flutamide) plus ADT. ENZA was associated with better OS (HR 0.43, 95% CI 0.26–0.72) and PFS (HR 0.30, 95% CI 0.22–0.43) among men with low-volume disease [7].

Similar to previous results with ENZA and AAP, hormonal intensification with APA in the TITAN trial [8], [9] delayed disease progression (radiographic PFS [rPFS]: HR 0.37, 95% CI 0.22–0.57) and was associated with better OS (HR 0.52, 95% CI 0.35–0.79) among men with low-volume mHSPC. While there was a clear statistical median OS benefit for men with high-risk disease (HR 0.57, 95% CI 0.45–0.73), OS outcomes favored the use of APA in low-risk disease but did not achieve statistical significance (HR 0.76, 95%CI 0.54–1.07).

In addition, ongoing trials are investigating other opportunities for hormonal intensification in the prostate cancer continuum, including patients with biochemical recurrence (EMBARK) and concomitant triple-agent intensification, such as the PEACE-1 [10] and ARASENS [11] trials. PEACE-1 is investigating the combination of standard-of-care treatment (ADT or ADT + docetaxel) ± radiotherapy and ± AAP for men with de novo mHSPC [10]. Its unique 2 × 2 factorial design allows assessment of triplet therapy (ADT + docetaxel + AAP) between groups with high-volume and low-volume disease. Men with high-volume disease benefited considerably from triplet therapy, with statistically better rPFS (HR 0.47, 95% CI 0.30–0.72) and OS (HR 0.72, 95% CI 0.55–0.95) in comparison to men with low-volume disease (rPFS: HR 0.58, 95% 0.29–1.15; OS: HR 0.83, 95% CI 0.50–1.39). The ARASENS trial is assessing a different triplet combination therapy (ADT + docetaxel ± darolutamide) for men with mHSPC [11]. While data comparing low-volume to high-volume disease have not yet been analyzed, the cohort experienced a striking improvement in OS (HR 0.68, 95% CI 0.57–0.80) and in the median time to castration resistance (HR 0.36, 95% CI 0.30–0.42) with the addition of darolutamide. Furthermore, the frequency of grade 3 or 4 adverse events was similar between the two groups (63.5% placebo vs 66.1% darolutamide) [11].

ADT monotherapy is no longer the preferred strategy for men with mHSPC. The data on dual and triplet hormonal intensification for men with mHSPC demonstrate clear improvements in oncologic outcomes, including survival, disease progression, time to pain, symptomatic skeletal events, and time to castration resistance, while patient quality-of-life parameters were maintained [5], [7]. Importantly, shared decision-making with patients in the early stage of metastatic disease is essential, including a discussion of alternatives to hormone intensification such as stereotactic body radiation therapy to low-volume metastatic sites via a clinical trial or primary prostate cancer radiation for low-volume mHSPC [12]. It must be recognized that the ongoing and future applicability of therapeutic regimens and the definition of low-volume mHSPC may change as more accurate imaging technology such as prostate-specific membrane antigen positron emission tomography becomes more accessible.

  ***Conflicts of interest*:** Seyed Behzad Jazayeri, Lauren Folgosa Cooley, and Abhishek Srivastava have nothing to disclose. Neal Shore has a consulting/advisory board relationship with AbbVie, Astellas, AstraZeneca, Bayer, Janssen, Merck, Myovant, Pfizer, and Tolmar.
